# Recovery of Physical Function during Hospitalization among Heart Failure Patients with and without Cachexia

**DOI:** 10.1298/ptr.25-E10364

**Published:** 2025-11-14

**Authors:** Takuya UMEHARA, Akinori KANEGUCHI, Yuji NAKASHIMA, Yosuke YAMAMOTO, Nobuhisa KATAYAMA, Nobuhiro KITO

**Affiliations:** 1 Department of Rehabilitation, Faculty of Rehabilitation, Hiroshima International University, Japan; 2 Department of Rehabilitation, Kure Kyosai Hospital, Japan

**Keywords:** Heart failure, Cachexia, Recovery of function

## Abstract

**Objectives:**

This study aimed to clarify the impact of cachexia on physical function recovery during hospitalization among patients with heart failure using the new Asian Working Group for Cachexia criteria and to identify the characteristics of heart failure patients with cachexia.

**Methods:**

Cachexia at discharge was defined by low body mass index combined with one or more of the following: low handgrip strength, elevated C-reactive protein, or anorexia. Physical function was assessed at admission and discharge. Two-way analysis of variance (ANOVA) was performed to examine the interaction and main effects of the presence of cachexia and duration factors (admission and discharge) on physical function. Hierarchical logistic regression analysis was performed to explore factors associated with the presence of cachexia.

**Results:**

Of the 96 patients analyzed, 26 were heart failure patients with cachexia, and 70 were heart failure patients without cachexia. The results of the 2-way ANOVA indicated that heart failure patients without cachexia exhibited improved physical function at discharge compared to that at admission. In contrast, heart failure patients with cachexia showed no improvement in physical function during hospitalization. Hierarchical logistic regression analysis revealed that a low geriatric nutritional risk index (GNRI) and low muscle power were associated with the presence of cachexia in patients with heart failure.

**Conclusions:**

Our results suggest that heart failure patients with cachexia experience poor recovery of physical function during hospitalization, and that reduced muscle power and deterioration in nutritional status, as indicated by a low GNRI, were associated with the presence of cachexia.

## Introduction

As populations age, the number of patients with heart failure in developed countries, such as Japan, has continued to increase^1)^. Heart failure leads to various issues, including heightened mortality rates, decreased quality of life, and an increased healthcare burden^2)^. Furthermore, heart failure has been known to cause weight loss, muscle mass reduction, chronic inflammation, malnutrition, and extreme fatigue, which promote the development of cachexia^3,4)^. Cachexia, characterized by involuntary weight loss, has been observed in patients with various chronic diseases, including heart failure^5)^. Moreover, this condition has been identified as a significant independent risk factor for hospital readmissions^6)^ and increased mortality rates due to heart failure^7)^. Therefore, investigating the pathophysiological mechanisms of cachexia in patients with heart failure is crucial.

Previous studies have reported that heart failure patients with cachexia have poorer physical function than those without cachexia^8–11)^. Hence, elucidating the characteristics of heart failure patients with cachexia is of considerable importance, given their potential to develop adverse events due to the decline in physical function. Moreover, cachexia, which involves chronic diseases along with muscle mass loss, chronic inflammation, and malnutrition, may limit responses to treatment. In fact, previous studies have reported that resistance training and aerobic exercise failed to improve muscle function or exercise tolerance in cancer patients with cachexia^12,13)^. However, the effects of cachexia on the recovery of physical function among patients with heart failure still remain poorly understood. Clarifying these effects would therefore provide valuable information for the management of such patients.

Recently, the Asian Working Group for Cachexia (AWGC) introduced a new definition for cachexia among patients with chronic diseases in Asia^14)^. Compared with the criteria proposed by the Cachexia Consensus Conference (Evans criteria), the AWGC criteria contain fewer items beyond the necessary requirements and utilize a different cutoff value for body mass index (BMI). Consequently, the AWGC criteria have the advantage of detecting cachexia earlier compared to the traditional Evans criteria. This study aimed to clarify the impact of cachexia on the recovery of physical function during hospitalization in patients with heart failure using the new AWGC criteria for cachexia and to identify the characteristics of heart failure patients with cachexia.

## Methods

### Study design

This prospective study was conducted in accordance with the principles outlined in the Declaration of Helsinki and the STROBE statement. Ethics approval was granted by the Research Ethics Committee of Kure Kyosai Hospital, Hiroshima, Japan (Ref. No. 2021-3) and Hiroshima International University, Hiroshima, Japan (Ref. No. 20-046).

### Setting

This study was conducted at a hospital. Recruitment, follow-up, and data collection were performed between February 2021 and September 2023. Potential patients were recruited by therapists affiliated with the rehabilitation department of the participating hospital.

### Patients

Heart failure was categorized as stage C or D based on the heart failure stage classification of the American College of Cardiology Foundation/American Heart Association^15)^. This classification is also consistent with the guidelines of the European Society of Cardiology^16)^ and the Japanese Circulation Society/Japanese Heart Failure Society (JCS/JHFS)^17)^. Patients hospitalized for acute heart failure treatment or acute exacerbation of chronic heart failure were included in this study. Only heart failure patients who were admitted were recruited, and outpatients were not included. Heart failure was diagnosed according to the Framingham criteria^18)^. The inclusion criteria were as follows: (1) age 65 years or older and (2) ability to walk independently upon admission. The exclusion criteria were as follows: (1) complications during admission, (2) presence of a pacemaker, and (3) diagnosis of severe dementia based on a score of 9 or lower on the revised Hasegawa Dementia Rating Scale. We excluded patients with pacemakers for 2 main reasons. First, the implantation of a pacemaker itself may affect exercise tolerance, autonomic regulation, and muscle function. A previous study has shown that cardiac pacing alters heart rate response and autonomic modulation during exercise, which could act as a confounding factor when evaluating physical function specifically attributable to heart failure^19)^. Second, bioelectrical impedance analysis (BIA), which we used to assess body composition, is contraindicated in patients with implanted devices such as pacemakers due to potential risks of device interference^20)^. Taken together, these considerations justify the exclusion of pacemaker patients in order to ensure both measurement validity and patient safety. We included patients with mild dementia because cognitive impairment is highly prevalent in patients with heart failure^21)^, and excluding them would limit the generalizability of our findings. Previous studies have shown that physical function assessments, such as gait speed and handgrip strength, can be reliably performed in patients with mild cognitive impairment^22)^. Cardiac rehabilitation interventions were implemented to address deconditioning and enhance activities of daily living during hospitalization. Safety measures focused on monitoring subjective symptoms and hemodynamic stability. Furthermore, blood pressure, SpO_2_, and electrocardiograms were continuously monitored during the rehabilitation sessions as part of risk management. The physical therapy program included aerobic exercises, such as walking, cycling on a bicycle ergometer, and resistance training targeting the lower limbs, tailored to each patient’s condition. Exercise intensity was determined using a comprehensive approach that incorporated target heart rate (calculated as resting heart rate plus 20 bpm), the talk test, and the Borg Rating of Perceived Exertion scale 11–13 for both the chest and legs. This method aligns with current guidelines and has been validated in various populations, including those with cardiovascular conditions. For instance, Reed and Pipe^23)^ discussed the use of these tools in prescribing and monitoring exercise intensity, emphasizing their practicality and effectiveness in clinical settings. Additionally, Eisenberger et al.^24)^ highlighted the correlation between the Borg Rating of Perceived Exertion scale and physiological measures such as heart rate and oxygen consumption during exercise. The type, duration, and intensity of exercise were progressively adjusted based on the patient’s condition.

### Sample size

The sample size was calculated using MedCalc Statistical Software version 19.2 (MedCalc Software bvba, Ostend, Belgium). A previous study comparing exercise function between heart failure patients with and without cachexia revealed a patient distribution of 1:2.5, respectively^25)^. Hence, our calculation used a 1:2.5 positive–negative ratio. The alpha level was set at 0.05, whereas the power was set at 0.9. The area under the receiver-operating characteristic curve (AUROC) was used to assess predictive ability, with the following classifications: non-predictive (AUROC <0.5), less predictive (0.5 <AUROC <0.7), moderately predictive (0.7 <AUROC <0.9), highly predictive (0.9 <AUROC <1), and perfectly predictive (AUROC = 1)^26)^. The AUROC for this hypothesis was set at 0.8 (indicating moderate power), whereas that for the null hypothesis was set at 0.5 (indicating no discriminatory power). Consequently, our calculation determined that the current study required 20 and 60 heart failure patients with and without cachexia, respectively (a total of 80 cases).

### Assessments of cachexia

Cachexia was evaluated using the AWGC criteria^14)^, which define cachexia based on (1) a BMI of <21 kg/m^2^ or weight loss exceeding 2% over 3–6 months, and (2) the presence of one or more of the following conditions: low handgrip strength (<28 for men and <18 kg for women), high C- reactive protein levels (>0.5 mg/dL), or anorexia. One of the symptoms of heart failure is edema, with patients typically experiencing weight loss due to treatment during hospitalization. Therefore, this study did not include treatment-induced weight loss in the determination of cachexia. Anorexia was defined as restricted dietary intake (<70% of normal dietary intake) or as determined by the registered dietitian. The presence of cachexia was determined at the time of discharge.

### Outcomes

Basic outcomes included age, sex, BMI, living together with family, length of hospital stay, degree of sarcopenia, and degree of frailty. The degree of frailty was assessed according to the Japanese version of the Cardiovascular Health Study Index criteria^27)^. The degree of sarcopenia was assessed according to the Asian Working Group for Sarcopenia^28)^. Degrees of sarcopenia and frailty were assessed at discharge, while other characteristics were determined upon admission.

Medical outcomes included the presence or absence of pharmacotherapy (dopamine, dobutamine, noradrenaline, phosphodiesterase III inhibitors, diuretics, and beta-blockers), the New York Heart Association classification scores, and the presence or absence of medical history (heart failure, coronary artery disease, valvular disease, hypertension, diabetes mellitus, dyslipidemia, atrial fibrillation, chronic renal failure, and stroke), as well as blood data (geriatric nutritional risk index [GNRI], brain natriuretic peptide, estimated glomerular filtration rate, hemoglobin, and C- reactive protein levels), and left ventricular ejection fraction. New York Heart Association classification scores, presence or absence of medical history, left ventricular ejection fraction, and blood data were measured upon admission, whereas C-reactive protein levels were measured at both admission and discharge. Medical history was obtained prior to hospitalization. For example, the absence of heart failure in the medical history indicated that no history of heart failure was present prehospitalization.

The extracellular water/total body water ratio and skeletal muscle mass index were measured using direct segmental multifrequency BIA (S10; InBody, Tokyo, Japan) in the supine position. BIA was performed using an 8-point tactile electrode system, with 30 impedance measurements taken at 6 different frequencies (1, 5, 50, 250, 500, and 1000 kHz). The tool was not statistically corrected for age, sex, or race during body water and skeletal muscle mass measurements and calculated extracellular water and extracellular water/total body water using a formula coded into the software based on measured height. The skeletal muscle mass index was calculated using the following formula: appendicular skeletal muscle mass/(height)^2^ (kg/m^2^). The extracellular water/total body water ratio and skeletal muscle mass index were measured at discharge.

Physical function was assessed using the short physical performance battery, handgrip strength, walking speed, and muscle power. The short physical performance battery is an established standardized and reproducible measure of motor function among older patients and comprehensively evaluates motor function based on 3 components: static standing balance, 4-m walk time, and time to complete 5 repeated chair stands. Each component is scored from 0 to 4 points, with higher scores indicating better physical function^29)^. Handgrip strength was assessed using a grip strength meter (TKK-5101; Takei Scientific Instruments, Tokyo, Japan) with the patients in the standing position and the upper limb abducted to approximately 20°^30)^. Measurements were performed twice for both sides, with the maximum value being adopted as the representative value. To assess walking ability, walking speed was measured at a pace comfortable for the patients^31)^. Measurements were performed twice at an interval of 30s, with the faster value being used to indicate walking speed. Muscle power was calculated based on the time patients took to complete 5 chair rises, in accordance with the report by Alcazar et al.^32)^. Weight, height, and chair height were also used to calculate muscle power using the following formula: weight × 0.9 × 9.8 × (height × 0.5 − chair height)/5 chair rises × 0.1. Muscle power is defined as force × velocity. We focused on muscle power because it has been reported to decline earlier than muscle strength in elderly individuals^33)^. Furthermore, reduced muscle power strongly predicts slower walking speed, impaired balance, decreased ability to perform activities of daily living, and progression of frailty^33)^. Physical function was measured upon admission and at discharge.

### Statistical analysis

Patient characteristics were compared using the Mann–Whitney U-test, t-test, or chi-squared test, as appropriate. Two-way analysis of variance (ANOVA) was performed to examine the interaction and main effects of the presence of cachexia and duration factors on physical function. Duration factors included admission and discharge. If significant interaction and/or main effects were detected, Bonferroni post hoc tests were performed to localize the effects. Variables considered significant for the basic outcomes were considered as covariates. Hierarchical logistic regression analysis was performed to explore the association between heart failure with cachexia (scored as 1) and heart failure without cachexia (scored as 0). Basic and medical outcomes, extracellular water/total body water, skeletal muscle mass index, and physical function were taken as the independent variables. Hierarchical logistic regression analysis was used in the 2 models: one that predicted cachexia at discharge from outcomes at admission, and the other that predicted cachexia at discharge from outcomes at discharge. First, statistically significant variables for the basic outcome were forcibly input as confounding factors into block 1. Thereafter, other independent variables were input into block 2 using a stepwise method. To account for multicollinearity, the correlation coefficient threshold between independent factors was set at 0.8, subsequently selecting those highly correlated with the dependent variable. BMI was not included as a covariate in the 2-way ANOVA and hierarchical logistic regression analysis, given our assumption of a statistically significant difference in BMI due to the cachexia criteria. All statistical analyses were performed using SPSS version 29.0 for Windows (IBM, Armonk, NY, USA), with a P value of <0.05 indicating statistical significance.

## Results

**Fig. 1.**
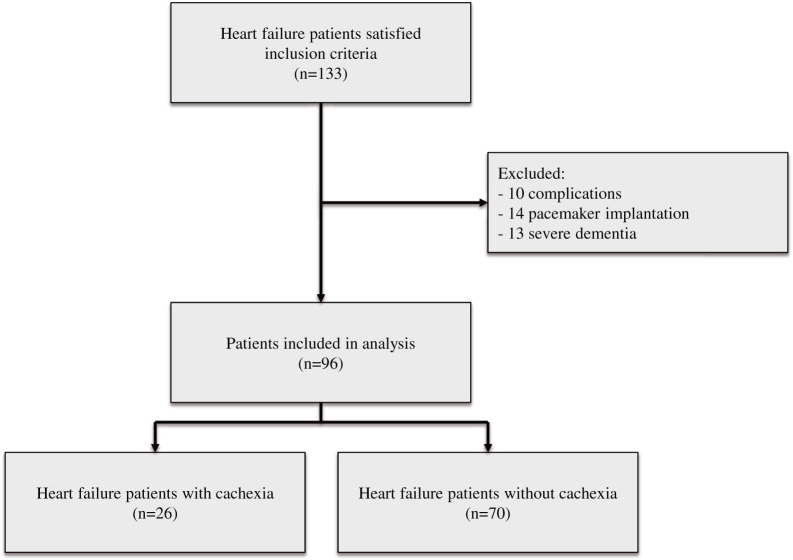
Flowchart for selecting patients

Figure 1 shows the flowchart for selecting patients. A total of 133 heart failure patients met the inclusion criteria. Among them, 10 patients experienced complications during admission, 14 underwent pacemaker implantation, and 13 had severe dementia. Ultimately, 96 patients were included in the analysis. Among the 96 patients analyzed, 26 had cachexia, whereas 70 did not. Table 1 summarizes the characteristics of heart failure patients with and without cachexia. Heart failure patients with cachexia were older, had a lower BMI and skeletal mass index, and had worse GNRI and skeletal muscle mass index than those without cachexia. No other variables were statistically significant.

**Table 1.** Patient characteristics

Time	Variable	Category	Heart failure patients without cachexia (n = 70)	Heart failure patients with cachexia (n = 26)	P value
Admission	Age (years)		83.00 (76.00–86.00)	86.00 (82.00–91.00)	0.03^‡^
	Sex	Female/male	35/35	13/13	1.00^†^
	BMI (kg/m^2^)		24.14 (22.77–25.31)	19.34 (18.28–19.79)	<0.01^‡^
	Family living together	Presence/absence	19/51	9/17	0.47^†^
	New York Heart Association (score)	1/2/3/4	3/17/35/15	0/10/6/10	0.93*
	Medical history				
	Heart failure	Presence/absence	46/24	14/12	0.28^†^
	Coronary artery disease	Presence/absence	17/53	7/19	0.79^†^
	Valvular disease	Presence/absence	18/52	7/19	0.91^†^
	Hypertension	Presence/absence	64/6	24/2	0.89^†^
	Diabetes mellitus	Presence/absence	24/46	6/20	0.29^†^
	Dyslipidemia	Presence/absence	29/41	9/17	0.54^†^
	Chronic renal failure	Presence/absence	21/49	7/19	0.76^†^
	Stroke	Presence/absence	15/55	4/22	0.51^†^
	Pharmacotherapy				
	Dopamine	Presence/absence	0/70	0/26	—
	Dobutamine	Presence/absence	3/67	0/26	0.28^†^
	Noradrenaline	Presence/absence	0/70	0/26	—
	Phosphodiesterase III inhibitor	Presence/absence	3/67	0/26	0.28^†^
	Diuretic	Presence/absence	23/47	7/19	0.51^†^
	Beta-blocker	Presence/absence	30/40	15/11	0.19^†^
	GNRI (score)		106.93 (98.75–126.05)	92.31 (83.44–117.46)	<0.01^‡^
	BNP (pg/ml)		418.10 (245.55–829.32)	714.25 (363.50–1241.20)	0.06^‡^
	eGFR (ml/min/1.73 m^2^)		42.75 (28.12–56.57)	40.20 (31.15–68.00)	0.31^‡^
	Hb (g/dl)		11.83 ± 2.03	11.15 ± 2.23	0.16*
	CRP		0.31 (0.19–0.80)	0.22 (0.09–1.11)	0.48^‡^
	LVEF (%)		52.05 (35.02–66.90)	41.60 (27.20–65.20)	0.18^‡^
	HDS-R (score)		25.00 (20.00–27.00)	22.00 (18.00–25.00)	0.08^‡^
	LSA (score)		48.00 (25.75–63.25)	36.00 (22.87–52.87)	0.16^‡^
	Length of stay (days)		15.00 (13.00–18.00)	16.00 (14.00–22.00)	0.20^‡^
	SMI		5.93 ± 1.08	5.19 ± 0.79	<0.01*
	ECW/TBW		0.40 (0.39–0.41)	0.40 (0.39–0.41)	0.63^‡^
Discharge	Degree of sarcopenia at discharge	Non-sarcopenia/sarcopenia/severe sarcopenia	2/10/14	34/13/23	0.76^†^
	Degree of frailty at discharge	Non-frailty/pre-frailty/frailty	3/10/13	9/27/34	0.98^†^

Average and standard deviation or median (interquartile range).

*Independent t-test.

^†^ Chi-squared test.

^‡^Mann–Whitney U-test.

BMI, body mass index; GNRI, geriatric nutritional risk index; BNP, brain natriuretic peptide, eGFR, estimated glomerular filtration rete; Hb, hemoglobin; CRP, C-reactive protein; LVEF, left ventricular ejection fraction; HDS-R, hasegawa dementia rating scale-revised; LSA, life space assessment; SMI, skeletal mass index; ECW, extracellular water; TBW, total body water

Table 2 presents the results of comparing physical function according to the presence of cachexia and duration factors. Accordingly, we found a significant difference in muscle power between the groups. Post hoc analyses showed that heart failure patients with cachexia had significantly worse muscle power than those without cachexia at both admission and discharge. Moreover, significant differences in short physical performance battery, 10-m gait speed, and muscle power were observed across duration factors. Post hoc analyses indicated that heart failure patients without cachexia showed significantly greater improvements in short physical performance battery, 10-m gait speed, and muscle power at discharge than at admission, whereas those with cachexia showed no significant improvements. No other variables were statistically significant.

**Table 2.** The results of the comparison of interaction and main effects of duration and between groups

Variable	Admission		Discharge		P value
	Heart failure patients without cachexia (n = 70)	Heart failure patients with cachexia (n = 26)	Heart failure patients without cachexia (n = 70)	Heart failure patients with cachexia (n = 26)	
SPPB (score)	7.76 ± 2.46	6.77 ± 2.77	9.24 ± 2.36^a^	8.42 ± 2.61	Duration <0.01 Between group 0.35 Interaction 0.81
10-m gait speed (seconds)	14.36 ± 4.85	15.43 ± 5.61	12.19 ± 3.78^a^	13.25 ± 4.09	Duration <0.01 Between groups 0.73 Interaction 0.99
Hand grip (kg)	20.24 ± 7.94	20.83 ± 7.85	18.51 ± 5.61	17.60 ± 5.28	Duration 0.87 Between groups 0.42 Interaction 0.47
Muscle power	136.65 ± 65.94	92.40 ± 50.26^b^	157.08 ± 73.96^a^	113.68 ± 53.51^b^	Duration <0.01 Between groups <0.01 Interaction 0.96

Average and standard deviation.

^a^Significant difference compared with admission (P < 0.05).

^b^Significant difference compared with patients without cachexia at the same time period (P <0.05).

SPPB, short physical performance battery

Table 3 presents the results of the hierarchical logistic regression analysis examining the association between heart failure and cachexia. Regarding the prediction of cachexia at discharge from the time of admission, the final independent variables included basic and medical outcomes, as well as physical function at admission. Our results revealed that GNRI and muscle power were significant predictors of cachexia (odds ratios, 0.97 and 0.99, respectively). Regarding the prediction of cachexia at discharge from the time of discharge, the final independent variables included basic and medical outcomes, extracellular water/total body water, skeletal muscle mass index, and physical functions at discharge. Accordingly, our results revealed that GNRI and muscle power were significant predictors of cachexia (odds ratios, 0.98 and 0.97, respectively).

**Table 3.** Logistic regression analysis results of the variable that affected the cachexia with heart failure patients

Time	Variable	Regression coefficient	Odds ratio	95% confidence interval		P value
				Lower limit	Upper limit	
Admission	Age	0.021	1.021	0.939	1.111	0.622
	GNRI	−0.030	0.971	0.947	0.995	0.019
	Muscle power	−0.012	0.988	0.976	0.999	0.037
	Constant	1.914				
Discharge	Age	−0.010	0.990	0.833	1.176	0.905
	GNRI	−0.206	0.814	0.689	0.961	0.015
	Muscle power	−0.035	0.966	0.934	0.999	0.042
	Constant	24.562				

Admission: χ^2^ test: P <0.05; discrimination rate = 81.1%.

Discharge: χ^2^ test: P <0.05; discrimination rate = 86.8%.

GNRI, geriatric nutritional risk index

## Discussion

The current study aimed to clarify the impact of cachexia on the recovery of physical function during hospitalization in patients with heart failure and to identify the characteristics of heart failure patients with cachexia. Two-way ANOVA revealed that heart failure patients without cachexia exhibited better improvements in physical function (i.e., short physical performance battery, 10-m walking speed, and muscle power) at discharge than at admission. In contrast, heart failure patients with cachexia demonstrated no improvement in physical function during hospitalization. Furthermore, hierarchical logistic regression analysis revealed that a low GNRI and decreased muscle power were associated with the presence of cachexia among heart failure patients. Our results suggest that heart failure patients with cachexia, who have deteriorated nutritional status and reduced muscle power, experienced poor recovery of physical function during hospitalization.

Two-way ANOVA revealed that heart failure patients with cachexia showed no improvement in physical function (i.e., short physical performance battery, 10-m walking speed, handgrip strength, and muscle power) at discharge when compared to that at admission. In contrast, heart failure patients without cachexia showed improvement in 3 out of 4 of these physical function measures (i.e., short physical performance battery, 10-m walking speed, and muscle power) during hospitalization. Considering that no previous studies have investigated the effects of cachexia on the recovery of physical function during hospitalization, our results present new evidence. Cachexia is a condition characterized primarily by irreversible muscle mass loss, persistent inflammation (promoting muscle protein breakdown and inhibiting recovery of muscle strength through exercise), energy deficiency, and deterioration of the overall systemic condition^5)^. Given the pathophysiology of cachexia as described earlier, heart failure patients with cachexia understandably experience difficulty in recovering their physical functions during hospitalization. However, a previous study has reported that exercise and nutritional therapies could improve muscle atrophy in heart failure patients with cachexia^34,35)^. This particular study included patients who were under 55 years of age, and the intervention periods were longer than 1 month. Alternatively, our study focused on elderly patients (≥65 years) who would be expected to be less responsive to exercise interventions, with a length of stay (intervention period) of approximately 15 days. These factors might explain why our heart failure patients with cachexia showed no improvement in physical function during hospitalization. Our 2-way ANOVA revealed no significant differences in the short physical performance battery, 10-m walking speed, and handgrip strength between heart failure patients with and without cachexia, both at admission and discharge. In contrast, a previous study found that heart failure patients with cachexia had significantly worse handgrip strength, walking speed, and short physical performance battery than did those without cachexia^36)^. This particular study focused on outpatient heart failure patients who were younger (81 ± 6.7 and 77 ± 8.5 years old in the cachexia and non-cachexia groups, respectively) than those included in our study, whereas the current study focused on heart failure patients in their late 80s who were hospitalized for acute exacerbation. Hence, even heart failure patients without cachexia experienced a decline in physical function, which might have complicated the detection of differences between those with and without cachexia.

Hierarchical logistic regression analysis revealed that low GNRI was associated with the presence of cachexia in heart failure patients. Indeed, 1 study identified malnutrition as a cause of cachexia^37)^. Malnutrition causes anabolic resistance and increased energy expenditure, leading to a gradual decrease in skeletal muscle mass^38)^. In the current study, heart failure patients with cachexia had a median GNRI of 92.31, indicating mild malnutrition, whereas those without cachexia had a median GNRI of 106.93, indicating no risk of malnutrition. Evidence has shown that heart failure is associated with a high risk of malnutrition^39)^. The causes of malnutrition among patients with heart failure are believed to be related to metabolic abnormalities induced by the pathophysiology of heart failure and its symptoms and signs^40)^. The former is a condition characterized by a sustained increase in energy expenditure, which depends on the duration and severity of heart failure^41)^, whereas the latter is influenced by heart failure itself, with its effects being particularly prominent during the acute phase^42)^. The current study was unable to conduct a detailed investigation on the former. For the latter, however, our findings showed that heart failure patients with cachexia tended to have higher levels of brain natriuretic peptide, which represents the severity of heart failure, than those without cachexia, albeit not significantly (cachexia group: 714.25 [363.50–1241.20], non-cachexia group: 418.10 [245.55–829.32], P = 0.06). In other words, heart failure by itself might contribute to malnutrition, which in turn may have triggered the development of cachexia. Our findings indicate that a low GNRI is associated with the presence of cachexia in patients with heart failure. For physical therapists, this suggests that assessment of nutritional status should be integrated into routine evaluations for hospitalized patients with heart failure. Early identification of patients with poor nutrition may allow targeted interventions, such as combining exercise therapy with nutritional support, to prevent further decline in physical function and promote recovery. Nutritional optimization may enhance the efficacy of physical therapy by mitigating muscle mass and strength loss associated with cachexia.

Two-way ANOVA revealed that heart failure patients with cachexia had significantly lower muscle power than did heart failure patients without cachexia at both admission and discharge. Meanwhile, hierarchical logistic regression analysis showed that low muscle power was associated with the presence of cachexia in heart failure patients. Muscle power is determined based on muscle strength and muscle contraction velocity^43)^, with a decrease in either or both of these factors leading to a reduction in muscle power. Evidence has shown that a decrease in muscle contraction velocity generally occurs due to a reduction in type II fast-twitch muscle fibers^44)^. Type II muscle fibers have been known to decrease with aging; however, individuals with cachexia demonstrate a more selective reduction in these fibers than did those without cachexia^9–11)^. Thus, heart failure patients with cachexia might experience a greater reduction in type II muscle fibers compared with those without cachexia, leading to decreased muscle contraction speed and potentially contributing to reduced muscle power. In a previous study, heart failure patients with cachexia displayed lower muscle strength than did those without cachexia^36)^. Furthermore, as described earlier, cachexia gradually reduces skeletal muscle mass due to metabolic abnormalities^38)^. In fact, our study showed that heart failure patients with cachexia had a significantly worse skeletal muscle mass index than did those without cachexia. Skeletal muscle mass index, an indicator of skeletal muscle mass, has been correlated with muscle strength^45)^. Therefore, both a decrease in muscle contraction speed and a reduction in muscle mass might contribute to the decline in muscle power through reduced muscle strength. However, the current study showed no significant difference in handgrip strength, which is considered an indicator of overall muscle strength, between patients with and without cachexia. A previous study found that age-related muscle strength decline occurs significantly more frequently in the lower limbs than in the upper limbs^46)^. Future studies are needed to investigate whether cachexia affects lower limb muscle strength in heart failure patients.

In our study, among 96 patients with heart failure, 21.0% had cachexia, whereas a previous study reported a prevalence of 74%^47)^. Although the 2 studies shared similar age, diagnostic criteria, and disease severity, several factors may account for the discrepancy in prevalence. First, differences in clinical backgrounds, such as comorbidities, nutritional status, and reasons for hospitalization, may have influenced the results. Second, the previous study was a large multicenter cohort involving more than 1000 patients, whereas our study was a smaller single-center cohort of 96 patients, which may have increased variability in patient composition. Third, advances in pharmacological treatment and inpatient management, including diuretics, novel heart failure drugs, and nutritional support, may have contributed to a lower prevalence of cachexia in our population at discharge. Therefore, our results differ from those of large-scale studies, and caution is required when generalizing the findings. However, in the FRAGILE-HF cohort study^25)^, the prevalence of cachexia was comparable to that in our study (35.4%). We assessed cachexia at the time of discharge to evaluate the overall impact of hospitalization on the development or progression of cachexia. We acknowledge that assessing cachexia at discharge may potentially reflect a failure of physical function recovery rather than pre-existing cachexia. However, by including patients who were independently ambulatory at admission, we aimed to focus on cachexia that likely developed or worsened during hospitalization. This approach allows for the identification of patients at risk for poor functional recovery and highlights the need for interventions targeting both nutrition and physical activity during hospitalization.

The current study has some limitations worth noting. First, cachexia was assessed at discharge considering that heart failure patients experience increased C-reactive protein levels and an increase in body weight due to edema associated with congestion during acute exacerbations, which complicates the accurate assessment of cachexia at admission. Additionally, while we assessed cachexia at discharge, it remains unclear whether cachexia developed during hospitalization or was already present prior to admission. In the future, if cachexia can be assessed upon admission, it could serve as a predictor of differences in subsequent physical function. Second, this study population included heart failure patients with a wide range of conditions, such as New York Heart Association classes I–IV at admission, and patients admitted for acute heart failure treatment or for acute exacerbation of chronic heart failure. These forms of heart failure differ in pathophysiology, exercise tolerance, and compensatory mechanisms, which may have introduced heterogeneity and potential bias into the interpretation of the results. Future studies should stratify patients by disease status and severity to allow more precise analyses. Third, although GNRI was identified as a significant result, albumin levels can be influenced by systemic inflammation during the acute phase. Elevated inflammatory markers such as C-reactive protein may lead to an overestimation of serum albumin levels, which in turn could have affected the accuracy of GNRI. Therefore, the interpretation of GNRI in acute settings should be made with caution. However, since admission C-reactive protein was included in our analyses and did not alter the main findings, we believe the likelihood of such overestimation influencing the results is low. In addition, there was no significant difference in admission C-reactive protein levels between the 2 groups, and the values were generally within the normal range, suggesting that marked systemic inflammation was unlikely to have affected our findings. Fourth, the AWGC criteria were used for assessing cachexia. Aside from the criteria used in the current study, the Cachexia Consensus Conference (Evans criteria) is another standard tool for assessing cachexia. A major difference between these criteria is the cutoff value for BMI. The AWGC criteria use a BMI of 21 kg/m^2^, whereas the Evans criteria use a BMI of 18.5 kg/m^2^. Given that BMI is an indicator of nutrition and can be used to infer muscle mass^48)^, this difference may have significantly affected the results of our study.

## Conclusions

Our results suggest that heart failure patients with cachexia experience poor recovery of physical function during hospitalization and that deterioration in nutritional status and reduced muscle power were associated with the presence of cachexia.
